# Public health round-up

**DOI:** 10.2471/BLT.16.010916

**Published:** 2016-09-01

**Breastfeeding “anytime, anywhere”**

Mother nurses her child in Santa Cruz, the Plurinational State of Bolivia. The theme of World Breastfeeding Week, from 1 to 7 August, was “support mums to breastfeed anytime, anywhere”.

**Figure:**
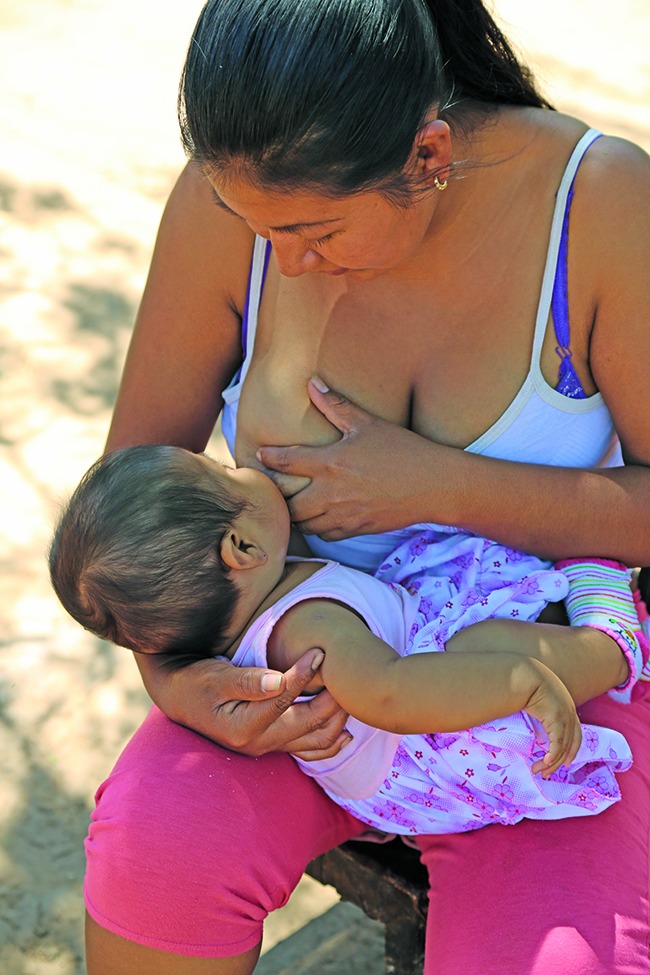
WHO/TDR /Fernando G Revilla

## UN meeting on antimicrobial resistance

Global leaders gather this month at the United Nations in New York to commit themselves to the fight against antimicrobial resistance (AMR) and decide on what action to take.

Antimicrobial resistance has become one of the greatest threats to global health and endangers other major priorities, such as human development.

Many common infections are becoming resistant to the antimicrobial medicines used to treat them, resulting in longer illnesses and more deaths; meanwhile too few new antimicrobial drugs, especially antibiotics, are being developed to replace older and increasingly ineffective ones.

The High Level Meeting on 21 September marks the fourth time in the history of the United Nations that a health topic is being discussed at the General Assembly. Previous such meetings were on HIV, noncommunicable diseases and Ebola.

Participants are expected to address the urgent need to take action and to agree on sustainable, multisectoral approaches to addressing the problem of antimicrobial resistance.

Last year the World Health Organization (WHO) released a report showing that only 34 of the 133 countries that responded to a WHO survey in 2013–2014 had comprehensive national plans to fight resistance to antibiotics and other antimicrobial medicines and even fewer already had systems in place to combat the problem.

The survey findings were presented in the report, *Worldwide country situation analysis: response to antimicrobial resistance*.

“Fortunately, Member States are making progress on their obligation to have national action plans in place by May 2017. The UN General Assembly meeting should result in high-level political commitment that will support countries in the global effort to stop AMR,” said Dr Keiji Fukuda, Special Representative of the Director-General on AMR.

http://www.who.int/drugresistance/events/UNGA-meeting-amr-sept2016/

## Cholera in South Sudan

A vaccination campaign got under way last month in South Sudan to stop a deadly outbreak of cholera. 

The health ministry, with support from WHO and other partners, has been ramping up disease surveillance, treatment and prevention efforts since July, when the cholera outbreak was confirmed. 

The campaign aims to reach more than 14 000 people with the oral cholera vaccine in the high risk areas of Juba county in the conflict-torn country, as it faces one of the world’s most severe humanitarian emergencies. 

A national cholera taskforce is coordinating efforts to stop the spread of the disease. 

It includes the health ministry, WHO, the United Nations Children’s Fund (UNICEF), Médecins Sans Frontières (MSF) and other health and water, sanitation and hygiene organizations that are part of the United Nations humanitarian cluster system. 

Cholera treatment centres have been set up at Juba Teaching Hospital and United Nations House civilian protection site by MSF, the health ministry and WHO; and 13 oral rehydration points have been established in Juba by Health Link South Sudan with support from partners including WHO to improve access to timely rehydration. 

WHO has distributed cholera preparedness and response guidelines to improve case detection and treatment of cholera.

Cholera is an acute diarrhoeal disease that causes massive loss of body fluids and can be deadly within hours if not adequately treated. 

“WHO is taking all the necessary control measures to support the Ministry of Health to respond to the situation urgently, and put an end to this outbreak,” said Dr Abdulmumini Usman, WHO Representative to South Sudan.

“This work is vital because the conditions are favourable for transmitting the disease. These include increased population displacement, overcrowding, unsafe water, poor hygiene and sanitation,” he said. 

WHO and partners are also supporting social mobilization and community engagement activities. The media is currently airing cholera prevention messages and a toll-free phone line to report cholera cases has been activated. 

http://who.int/emergencies/south-sudan

## Taking action on NCDs

A new WHO reports calls on governments to step up action to protect people from noncommunicable diseases (NCDs), especially heart disease, cancers, diabetes and lung diseases that are responsible for most deaths in people under 70 years of age.

According to the report entitled *Assessing national capacity for the prevention and control of noncommunicable diseases* some countries are making progress in moving closer to achieving the sustainable development goals and others are lagging behind.

The findings of the report are based on a survey to which 177 of WHO’s 194 Member States responded. Some 93% (164) of countries that responded said they had a unit, branch or department responsible for NCDs within their ministry of health and that 91% (161) had a full-time technical or professional staff member working on this. 

The report commended countries that have taken key public health measures, for example, to protect people from exposure to tobacco use, harmful use of alcohol, unhealthy diet and physical inactivity. 

The report also tracks progress on four time-bound commitments agreed in 2014 to strengthen countries’ abilities to tackle NCDs: to set national NCD reduction targets; to develop national multisectoral policies and plans to achieve these national targets; to reduce exposure to risk factors for NCDs; and to strengthen health systems to address NCDs.

To date, 60% (106) of countries have set national time-bound targets for NCD indicators and 92% (162) have integrated NCDs into their national health plans.

Respondents said that their top health funding priorities were health care and treatment (94%; 166 countries), followed by primary prevention of NCDs (88%; 156 countries) and early detection and screening (85%; 151 countries).

Just over a third (34%; 60) of countries reported having an operational national multisectoral commission, agency or mechanism to oversee NCD engagement, policy coherence and accountability of sectors beyond health, while 53% (93) of countries reported having an operational, multisectoral national policy, strategy or action plan that integrates several NCDs and their risk factors.

Nearly a quarter of countries (24%; 43) had not conducted a recent national adult risk factor survey.

http://www.who.int/ncds/events/action-on-ncds/

## Building laboratory capacity in Africa

A new international initiative was launched in July to make tuberculosis diagnostics more widely available in African countries, especially for HIV-associated and drug-resistant tuberculosis.

The burden of tuberculosis, including multidrug-resistant forms, is high, while the prevalence of tuberculosis and HIV co-infection is about 34% in southern and eastern African countries.

Laboratories need to be able to detect tuberculosis in all its forms rapidly and accurately, so that patients can be given prompt and appropriate treatment.

The Global Laboratory Initiative for Africa (GLI Africa) was launched to support countries in the WHO African Region to achieve quality-assured, accessible and sustainable tuberculosis laboratory services. 

This first GLI Africa workshop was organized in July in Uganda and brought together representatives of National Tuberculosis Control Programmes and National Tuberculosis Reference Laboratories from more than 20 African countries, technical partners, multilateral agencies, donors and other key stakeholders. 

The workshop identified key priorities for strengthening tuberculosis diagnostic networks in Africa and participants drafted the Kampala Declaration, which describes the next steps needed to strengthen tuberculosis diagnostic networks on the continent and affirms the role of GLI Africa as a partnership that can drive these steps forward.

Uganda has made considerable progress in building laboratory capacity in recent years.

The Uganda National TB Laboratory was accredited by WHO to become a supranational laboratory in 2013, giving the laboratory a mandate to support laboratory capacity building efforts in 11 African countries.

http://www.afro.who.int/en/uganda/press-materials/item/8847-global-laboratory-initiative-to-build-capacity-in-africa.html

**Cover photo**

This month’s cover photo shows cattle grazing in the United Republic of Tanzania. Methods of animal husbandry are changing, especially in low- and middle-income countries.

**Figure:**
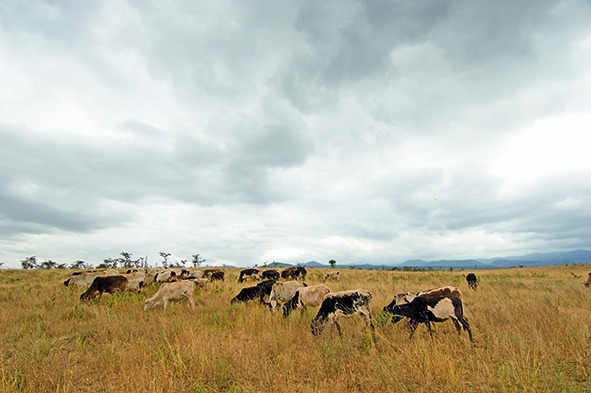
Wellcome Library, London

## Ending violence against children

WHO and partners have launched seven strategies to prevent violence against children and enhance victim services. 

The set of approaches launched in July, called INSPIRE, have all been tested and shown to be effective in different countries.

Each letter of INSPIRE corresponds to a strategy, with I for implementation and enforcement of laws; N for norms and values; S for safe environments; P for parent and caregiver support; I for income and economic strengthening; R for response and support services; and E for Education and life skills.

By bringing these seven approaches together in a package, WHO hopes that countries will adopt these to reduce violence against children.

Over the last year up to one billion children experienced physical, sexual or psychological violence, according to a recent study published in *Pediatrics*. 

Homicide is among the top five causes of death for adolescents. One in four children suffer physical abuse, and nearly one in five girls is sexually abused at least once in their lives.

www.who.int/violence_injury_prevention/violence/inspire

## New WHO emergencies chief

Dr Peter Salama has been appointed Executive Director in charge of WHO’s new Health Emergencies Programme and took up the new post in July. 

A medical epidemiologist from Australia, Salama was previously with UNICEF as Regional Director for the Middle East and North Africa and Global Emergency Coordinator for the crises in Syria, Iraq and Yemen.

Before that he worked at UNICEF, the United States Centers for Disease Control and Prevention, Concern Worldwide and MSF.

**Looking ahead**

**1–2 September – WHO-wide strategy meeting on migration and health, Venice, Italy.**

**11–12 September – G7 health ministers meeting in Kobe, Japan.**

**13–26 September – General Assembly of the United Nations.**

**19 September – United Nations Summit on Refugees and Migrants.**

**14–20 November – World Antibiotic Awareness Week.**

**1 December – World AIDS Day.**

